# Comparison of multiple machine learning methods for predicting postoperative hyperglycemia in patients without diabetes undergoing cardiac surgery

**DOI:** 10.3389/fcvm.2025.1699809

**Published:** 2025-11-27

**Authors:** Jinyan Wu, Mengli Zhang, Senxiu Cui, Guili Yang, Lulu Wang, Huan Duan, Fang Xue

**Affiliations:** 1 School of Nursing, Bengbu Medical University, Bengbu, Anhui, China; 2 Cardiovascular Medicine, The Third People's Hospital of Bengbu, Bengbu, Anhui, China; 3 Medical Oncology, First Affiliated Hospital of Bengbu Medical University, Bengbu, Anhui, China; 4 Cardiac Surgery, First Affiliated Hospital of Bengbu Medical University, Bengbu, Anhui, China; 5 Intensive Care Unit, First Affiliated Hospital of Bengbu Medical University, Bengbu, Anhui, China

**Keywords:** stress hyperglycaemia, model prediction, nomograms, risk assessment, retrospective analysis

## Abstract

**Background:**

Stress-induced hyperglycemia (SHG) represents a significant metabolic complication in non-diabetic cardiac surgery older adult patients, with substantial implications for postoperative outcomes. Despite its clinical importance, reliable predictive tools remain scarce. This study systematically compared the performance of logistic regression 5 s. advanced machine learning algorithms for SHG risk prediction in this vulnerable population.

**Patients and Methods:**

We conducted a retrospective cohort analysis of 600 patients (≥65 years) undergoing cardiac surgery at a tertiary medical center (January 2021–May 2025). Six clinically relevant perioperative variables were incorporated into five predictive models: logistic regression, Random Forest (RF), Gradient Boosting Machine (GBM), Adaptive Boosting (AdaBoost), and Extreme Gradient Boosting (XGBoost). Model performance was rigorously evaluated using AUC-ROC with 95% confidence intervals, sensitivity, specificity, positive (PPV) and negative predictive values (NPV), and precision.

**Results:**

The incidence of SHG in this cohort was 70.5%. Comparative analysis revealed logistic regression as the top-performing model (AUC 0.944, 95% CI 0.923–0.966), surpassing other algorithms: GBM (0.923, 0.902–0.952), 10GBoost (0.904, 0.890–0.941), AdaBoost (0.916, 0.871–0.936), and RF (0.877, 0.866–0.932). Moreover, the logistic model achieved optimal performance in sensitivity (94.5%), specificity (93.4%), PPV (97.7%), and NPV (96.8%).

**Conclusion:**

In contrast to more complex machine learning approaches, logistic regression demonstrated superior predictive accuracy for SHG in non-diabetic cardiac surgery older adult patients. Its exceptional performance metrics and clinical interpretability support its practical utility as an effective decision-support tool for perioperative risk stratification and management.

## 1 Introduction

Stress-induced hyperglycemia (SIH) following cardiac surgery presents a formidable clinical challenge, with reported incidence rates as high as 27.9% in cardiothoracic populations (1). Increasing evidence suggests that SIH is not merely a transient metabolic disturbance but a strong predictor of both acute complications—such as cardiogenic shock, life-threatening arrhythmias, and cerebrovascular events (2, 3)—and long-term mortality in patients without diabetes (4, 5). The stress hyperglycemia ratio (SHR) has demonstrated superior prognostic value than conventional glucose metrics for predicting adverse cardiovascular outcomes (4), with the first 48-h postoperative period representing a critical window for glycemic monitoring (6, 7). While current management protocols recommend maintaining glucose levels within 140–180 mg/dL (8–12), these reactive measures fail to address the pressing need for proactive risk identification, highlighting a key limitation and reinforcing the importance of predictive modeling in perioperative care.

Existing approaches to SIH prediction face several critical limitations that constrain their clinical utility. First, most models disproportionately focus on diabetic populations, overlooking the unique pathophysiology of non-diabetic individuals. Second, they often fail to integrate key metabolic-inflammatory markers, (e.g., C-reactive protein [CRP] and white blood cells [WBC], which significantly influence hyperglycemic responses (13, 14). Third, conventional statistical models lack the sophistication to capture complex clinical patterns, while machine learning alternatives are hampered by poor interpretability due to their reliance on statistically derived rather than clinically meaningful features (15). This “black box” problem presents particular challenges in surgical settings where transparent, actionable predictions are essential. The absence of comprehensive comparisons between traditional and advanced predictive methods further compounds these limitations.

Our research addresses these gaps through a systematic evaluation of logistic regression vs. contemporary machine learning algorithms [e.g., Extreme Gradient Boosting Machine [eGBM], Extreme Gradient Boosting [XGBoost], and Random Forest [RF]] for SIH prediction. This study employs an innovative approach that integrates three key elements: using clinically relevant variables with established pathophysiological significance; performing rigorous algorithmic comparison to optimize both accuracy [area under the curve (AUC) > 0.94] and clinical utility; and demonstrating that logistic regression provides a superior balance between performance and interpretability. The resulting model enables early identification of high-risk patients while maintaining clinical transparency, which is a crucial advantage over existing alternatives (16). By bridging the gap between computational sophistication and clinical applicability, this work represents a significant advancement in perioperative risk prediction and patient management.

## 2 Material and methods

### 2.1 Definition of stress hyperglycemia

SHG refers to a transient increase in blood glucose levels in individuals without a prior history of diabetes, occurring under conditions of severe stress such as major trauma, critical infections, or cardiovascular events. Prior to the stressor, the individual's blood glucose is completely normal; however, during the stress response, blood glucose levels significantly exceed the normal range. Once the stressor is removed, blood glucose levels typically return to normal. Currently, there is no standardized diagnostic criterion for stress hyperglycemia in China. The American Diabetes Association (ADA) defines the diagnostic criteria for SHG (17), which include fasting blood glucose levels ≥7.0 mmol/L on two or more occasions, or random blood glucose levels ≥11.1 mmol/L, in non-diabetic patients during periods of acute stress. In this study, to ensure consistency, all postoperative blood glucose measurements were obtained under fasting conditions in the morning according to the hospital's standardized testing protocol. Measurements were taken before meals and outside acute stress events to minimize confounding influences on glucose levels.

### 2.2 Measurement methods of stress hyperglycemia

The blood glucose levels were monitored using the Shengjia Steady Hao model rapid glucose meter to measure capillary blood glucose. According to the blood glucose monitoring guidelines reported in the literature (18, 19), when the patient's blood glucose concentration is 8.0 mmol/L or above, it is monitored every 0.5–1.0 h. When the blood glucose concentration is below 8.0 mmol/L, it is monitored every 2 h. Once the blood glucose results stabilize for 4 h, monitoring is performed every 4 h. Postoperatively, when blood glucose levels range from 8.0 mmol/L to 12.0 mmol/L, the glucose infusion rate is adjusted accordingly. If blood glucose levels exceed 12.0 mmol/L, insulin therapy is administered via intravenous micro-pump injection of recombinant insulin with a concentration ratio of 1:5–1:1. The initial dosage is 0.05 U/(kg·h) to 0.10 U/(kg·h), and the micro-pump speed is adjusted based on the blood glucose levels. The maximum insulin infusion rate is 0.5 U/(kg·h) (20).

### 2.3 Study endpoints

The occurrence of SHG after cardiac surgery was used as the outcome variable.

### 2.4 Setting

The 423 patients from January 2021 to 2024 May constituted the modeling group for internal validation, and the 177 patients from June 2024 to May 2025 constituted the validation group for external validation.

### 2.5 Study population

A retrospective collection of 600 patients treated at the First Affiliated Hospital of Bengbu Medical University between January 2021 to May 2025 was included in the study.

### 2.6 Inclusion and exclusion criteria

#### 2.6.1 Inclusion criteria

The study population comprised consecutive adult patients (≥18 years) who underwent elective or emergency cardiac surgical procedures at our tertiary referral center. Eligible surgical interventions included isolated coronary artery bypass grafting (CABG), valve replacement/repair procedures, and combined major cardiac vascular operations. Strict diabetes exclusion criteria were applied, requiring: (i) absence of documented diabetes mellitus in medical records or by patient self-report, (ii) confirmation of normoglycemic status per ADA standards (preoperative fasting plasma glucose <7.0 mmol/L and HbA1c<6.5%). All enrolled participants either provided written informed consent (prospective cohort) or had comprehensively documented medical records with institutional review board approval (retrospective cohort), with mandatory availability of serial postoperative glucose measurements (minimum 48 h monitoring period) for reliable assessment of stress-induced glycemic responses.

#### 2.6.2 Exclusion criteria

We applied rigorous exclusion parameters to ensure cohort homogeneity and data quality: (i) any preoperative diagnosis of diabetes mellitus or current antihyperglycemic therapy use; (ii) evidence of significant hepatic dysfunction (Child-Pugh class C cirrhosis) or end-stage renal disease (eGFR <30 mL/min/1.73 m^2^); (iii) preoperative systemic inflammatory conditions (sepsis, septic shock, or severe active infection); (iv) known secondary causes of glucose metabolism disorders (including paraneoplastic syndromes and endocrine disorders such as Cushing's syndrome or uncontrolled thyroid dysfunction); (v) postoperative mortality or study attrition within the initial 48-hour metabolic monitoring window; and (vi) inadequate glycemic monitoring (defined as either missing glucose values or insufficient measurement frequency to permit reliable assessment of glycemic variability).

### 2.7 Ethics statements

This study follows the principles of the Declaration of Helsinki and has been approved by the Ethics Committee of the First Affiliated Hospital of Bengbu Medical University [approval number (2024):KY012]. The research subjects understand the research purpose and collect data after obtaining informed consent. The research subjects may withdraw from the study at any time. The medical records of the research subjects are digitally encoded, stored anonymously and securely, and are only used for this study.

### 2.8 Model development and training strategy

In this study, five models—logistic regression (GLM), random forest (RF), gradient boosting machine (GBM), XGBoost, and AdaBoost—were constructed for comparison. To ensure fair comparison across models, all algorithms were trained using default parameter configurations without systematic hyperparameter tuning, except for the GBM model, in which the optimal number of trees was selected via fivefold cross-validation. This strategy minimized human-induced optimization bias, ensured consistent evaluation conditions, and maintained the stability and reproducibility of the results.

### 2.9 Model construction plan

In this study, we conducted a comparative evaluation of five machine learning algorithms—logistic regression (GLM), random forest (RF), gradient boosting machine (GBM), XGBoost, and AdaBoost. To ensure an unbiased comparison, all models were trained using their default parameter settings without systematic hyperparameter tuning, thereby minimizing the influence of manual optimization and enabling an objective assessment under consistent experimental conditions. A logistic regression model was employed to predict the outcome of stress hyperglycaemia. Patients were randomly divided into training and validation datasets at a 7:3 ratio. Variables showing significance in univariate analyses were included in the multivariable model, with forward and backward stepwise selection applied for variable refinement (21–24). Based on the resulting regression coefficients, an individualised nomogram was constructed to predict stress hyperglycaemia during major surgery. The model's performance was evaluated in the validation cohort using the area under the receiver operating characteristic curve (AUC). The remaining machine learning models—RF, GBM, AdaBoost, and XGBoost—were similarly developed to enable a comprehensive comparison of predictive performance.

### 2.10 Data collection

The study utilized comprehensive clinical data extracted from the hospital's electronic medical records system. To ensure clinical relevance and predictive validity, we systematically selected preoperative and intraoperative variables with established or plausible associations with stress-induced hyperglycemia. The collected parameters encompassed: (1) demographic and baseline clinical characteristics including age, sex, BMI, comorbidities (hypertension, prior cardiac surgery, cardiovascular disease, chronic obstructive pulmonary disease, chronic kidney disease, and cerebrovascular disease), ASA physical status classification, valvular heart disease, substance use history (smoking and alcohol consumption), hyperlipidemia, congestive heart failure, anemia, cardiogenic shock, recent myocardial infarction, aortic dissection, pulmonary disease, and advanced cardiac dysfunction (NYHA class ≥3); (2) preoperative medication exposure, particularly glucocorticoid administration (dexamethasone or methylprednisolone); (3) laboratory parameters including leukocyte count, serum uric acid, CRP, and renal function markers (with creatinine >200 μmol/L defined as clinically significant elevation); and (4) intraoperative variables consisting of surgical procedure type, operative duration, estimated blood loss, transfusion requirements (encompassing all blood product components), vasopressor use (norepinephrine), combined valve/CABG procedures, aortic cross-clamp time ≥110 min, reoperation status, prolonged cardiopulmonary bypass (>3 h), and CPB-associated hyperoxia.

### 2.11 Statistical and predictive modeling methodology

Statistical analyses were performed using a two-stage analytical framework. Initial univariate screening of potential risk factors was conducted in SPSS (v27.0), with statistically significant variables (*p* < 0.05) subsequently incorporated into multivariate logistic regression models after assessing multicollinearity through variance inflation factors (VIF <5 considered acceptable). For predictive modeling, the dataset underwent stratified random partitioning (70:30 training:validation ratio) to preserve outcome distribution. Five distinct algorithms—logistic regression, RF, GBM, AdaBoost, and XGBoost—were implemented in R (v4.2.2) using standardized preprocessing pipelines. Model performance was rigorously evaluated through: (1) internal 5-fold cross-validation within the training cohort, and (2) external validation using the hold-out set, with comprehensive metrics including AUC-ROC, sensitivity, specificity, PPV/NPV, and precision. Ensemble methods additionally underwent feature importance analysis to quantify predictor contributions, ensuring both predictive accuracy and clinical interpretability.

### 2.12 Data division and justification for selected predictive models in clinical settings

Total sample size: 600 non-diabetic patients undergoing cardiac surgery. Data partitioning: The dataset was randomly divided into a training (modeling) set (*n* = 423, 70%) and an independent validation set (*n* = 177, 30%).

Cross-validation: Within the training set only, five-fold cross-validation was used to train the models and tune internal parameters (e.g., number of trees in the GBM).The validation set remained completely independent and was not used during model training or cross-validation. A schematic flowchart of the data splitting and validation procedure has been added to the revised manuscript to illustrate this process more clearly.

**Figure 1:**
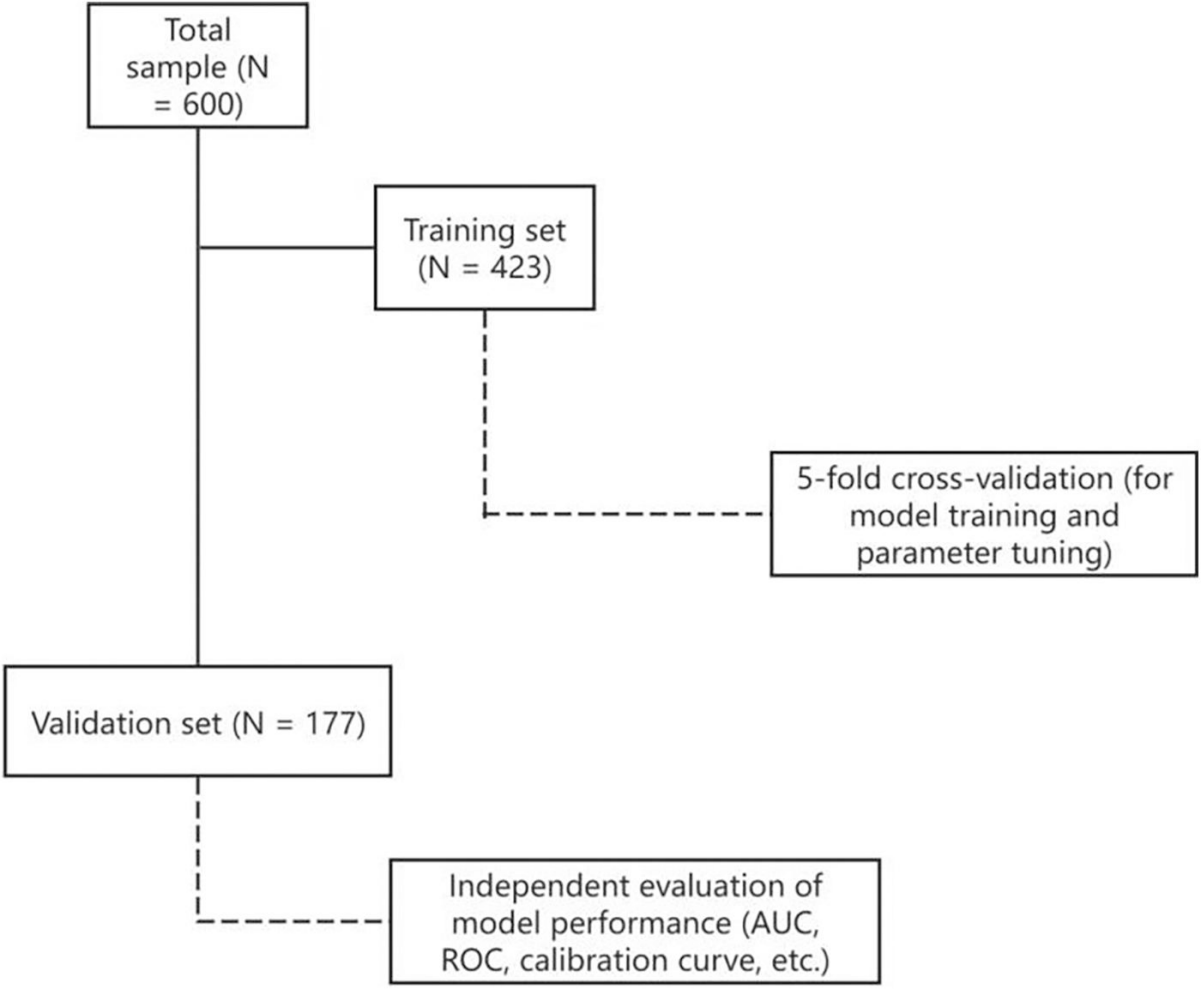
Data splitting and validation flowchart.

To ensure methodological rigor, we used stratified random sampling to split the dataset into training (70%) and validation (30%) sets (see Figure 1). This strategy maintained consistent distributions of both hyperglycemic (SIH) and normoglycemic (AH) cases across partitions, thereby minimizing sampling bias and preserving the clinical prevalence of the target condition. Within the training cohort, we employed k-fold cross-validation to enhance model generalizability and prevent overfitting. For predictive modeling, we strategically selected five established algorithms representing distinct methodological approaches: logistic regression provided a clinically interpretable parametric baseline, while four advanced ensemble methods—RF, GBM, AdaBoost, and XGBoost—were implemented to capture complex nonlinear relationships and interaction effects while maintaining interpretability through feature importance quantification. This comprehensive analytical framework enabled robust comparison of traditional statistical modeling with contemporary machine learning techniques, balancing predictive performance with clinical applicability.

### 2.13 Validation strategies

To ensure comprehensive evaluation of model performance, we implemented a tripartite validation strategy incorporating both internal and external validation methodologies. First, k-fold cross-validation was systematically employed, wherein the dataset was partitioned into k mutually exclusive subsets, with each subset serving sequentially as the validation set while the remaining k-1 subsets were used for model training. This approach effectively minimizes random sampling bias while providing robust estimates of model generalizability. Second, external validation was conducted using a temporally and geographically distinct patient cohort, offering critical assessment of real-world clinical applicability beyond the derivation dataset. Third, internal validation was performed through intensive resampling techniques, including both k-fold and leave-one-out cross-validation, enabling rigorous evaluation of model stability and reliability within the development cohort. This multi-dimensional validation framework provides complementary evidence of model performance across different clinical and methodological contexts.

### 2.14 Data preprocessing and model evaluation protocols

To ensure methodological rigor and reproducibility, we implemented a comprehensive data preprocessing and analytical pipeline comprising six key components: (1) missing data were addressed through multiple imputation by chained equations (MICE) to preserve dataset completeness while minimizing bias; (2) all continuous variables underwent z-score normalization to standardize feature scales, particularly critical for distance-sensitive algorithms (SVM, KNN) and linear models; (3) categorical variables were uniformly transformed using one-hot encoding to maintain consistency across model comparisons; (4) hyperparameter optimization was conducted via Bayesian optimization with Gaussian processes, enabling efficient identification of optimal parameter configurations for each algorithm; (5) model validation employed a stratified 5-fold cross-validation framework with maintained class distributions to robustly assess generalizability while mitigating overfitting; and (6) performance evaluation incorporated multiple complementary metrics including AUC-ROC, sensitivity, specificity, and precision-recall analysis to provide comprehensive assessment of model discrimination and calibration characteristics. This standardized protocol ensured consistent, reproducible model development while accounting for both predictive performance and clinical applicability.

### 2.15 Confounding control and adjustment

To minimize confounding in the SHG prediction model, we implemented a systematic approach encompassing strict cohort selection (non-diabetic adults >18 years undergoing cardiac surgery), prospective collection of key clinical and surgical variables (demographics, comorbidities, procedural details, and perioperative management), and rigorous analytical methods, including multivariable logistic regression with spline terms for non-linear effects, collinearity assessment, multiple imputation for missing data, and comprehensive sensitivity analyses (stratified models and penalized regression), thereby ensuring robust and clinically valid risk prediction.

### 2.16 Expected applications of the nomogram

This clinically validated nomogram provides four key utilities for perioperative management: (1) individualized risk quantification through weighted integration of demographic, preoperative, surgical, and postoperative parameters; (2) objective risk stratification (low/intermediate/high) to guide tiered intervention strategies—from intensive glucose monitoring/early insulin therapy for high-risk cases to standard surveillance for low-risk patients; (3) data-driven decision support that supplements clinical judgment for interventions, such as corticosteroid-associated glycemic control; and (4) optimized resource allocation by matching monitoring intensity and nursing care. The multidimensional scoring system of the tool balances precision with practicality in routine cardiac surgical practice.

### 2.17 Clinical application of the nomogram

The nomogram is implemented through a structured clinical workflow beginning with data acquisition, encompassing demographic characteristics (e.g., age, sex, BMI), preoperative comorbidities (e.g., hypertension, prior cardiac surgery), surgical variables (e.g., procedure type/duration, blood loss), and postoperative management (e.g., corticosteroid administration). Subsequently, risk quantification translates input variables into weighted scores via the algorithmic framework of the nomogram, with differential weighting reflecting each parameter's predictive contribution (e.g., corticosteroids >age). The aggregated scores are then converted into probabilistic estimates through validated calibration during risk projection (e.g., 0.25 = 25% SHG risk). Finally, risk-stratified management guides clinical actions: high-risk patients (≥50% probability) receive intensive monitoring and prophylactic insulin protocols, while low-risk patients undergo standard surveillance without additional interventions. This standardized yet adaptable protocol ensures reproducible risk assessment while preserving clinician discretion in therapeutic decision-making.

### 2.18 Missing data handling

All six predictive variables were first examined for missingness, and all were found to be complete (missing count = 0; missing rate = 0%). Consequently, multiple imputation by chained equations (MICE) was not applied. For studies with missing values, MICE can be used to impute variables under the assumption of missing at random, employing predictive mean matching for continuous variables and logistic regression for categorical variables. Typically, five imputed datasets are generated and analyzed separately, with final estimates pooled using Rubin's rules to account for variability between imputations. In the present study, logistic regression and all machine learning models (RF, GBM, XGBoost, AdaBoost) were trained and validated on the complete dataset, and all performance metrics, including ROC, AUC, and calibration, were calculated based on this dataset (25).

Assessment of model calibration using bootstrap resampling

## 3 Results

### 3.1 Patient characteristics

This retrospective cohort study evaluated 600 consecutive adult patients (>18 years) undergoing cardiac surgery at the First Affiliated Hospital of Bengbu Medical University between January 2021 and May 2025. Following rigorous application of inclusion/exclusion criteria with complete case analysis (no exclusions for missing data), the cohort was stratified into model development (*n* = 423) and validation (*n* = 177) groups. Postoperative stress-induced hyperglycemia (P-SIH) within 48–72 h occurred in 423 patients (70.5%), consistent with established metabolic stress responses following cardiac procedures. Analysis of P-SIH cases (*n* = 303) revealed male predominance (54.8% vs. 45.2% female) with primary surgical indications being valve procedures (49.2%) and coronary artery bypass grafting (39.9%). Key risk factors included advanced age (>65 years, 41.9%), obesity (BMI ≥28 kg/m^2^, 15.2%), and prevalent comorbidities, such as hypertension (53.5%), hyperlipidemia (41.3%), and congestive heart failure (22.4%). Notable metabolic markers included elevated uric acid (73.9%) and anemia (70.3%). Significant surgical factors comprised prolonged cardiopulmonary bypass (>3 h, 67%), extended aortic cross-clamp time (>90 min, 56.8%), and lengthy procedures (>5 h, 32.7%). Pharmacological contributors included norepinephrine administration (61.7%) and preoperative glucocorticoid use (79.5%), with high-risk patients (ASA ≥3: 6.3%; NYHA class ≥3: 32%) demonstrating particular susceptibility. The complete perioperative characteristics are detailed in Table 1.

**Table 1:** Patient characteristics and baseline variables.

Variables, *n* (%)	Category	AH (*n* = 120), *n* (%)	SIH (*n* = 303), *n* (%)	*P*-value
Sex	Male	72 (60.0)	166 (54.8)	0.38
	Female	48 (40.0)	137 (45.2)	
Age >65 years	No	83 (69.2)	176 (58.1)	0.04
	Yes	37 (30.8)	127 (41.9)	
Valvular heart disease	No	30 (25.0)	141 (46.5)	*P* < 0.0001
	Yes	90 (75.0)	162 (53.5)	
Heart valve surgery	No	37 (30.8)	154 (50.8)	*P* < 0.0001
	Yes	83 (69.2)	149 (49.2)	
Cardiac coronary artery bypass grafting	No	90 (75.0)	182 (60.1)	0.004
	Yes	30 (25.0)	121 (39.9)	
Great vascular surgery of the heart	No	116 (96.7)	293 (96.7)	0.986
	Yes	4 (3.3)	10 (3.3)	
Two kinds of operations	No	112 (93.3)	281 (92.7)	0.83
	Yes	8 (6.7)	22 (7.3)	
Three kinds of operations	No	118 (98.3)	301 (99.3)	0.335
	Yes	2 (1.7)	2 (0.7)	
BMI ≥ 28 Kg/m^2^n	No	108 (90.0)	257 (84.8)	0.163
	Yes	12 (10.0)	46 (15.2)	
Smoking history	No	26 (21.7)	67 (22.1)	0.92
	Yes	94 (78.3)	236 (77.9)	
Drinking history	No	95 (79.2)	253 (83.5)	0.293
	Yes	25 (20.8)	50 (16.5)	
History of cardiac surgery	No	109 (90.8)	271 (89.4)	0.669
	Yes	11 (9.2)	32 (10.6)	
Dialysis history	No	115 (95.8)	301 (99.3)	0.011
	Yes	5 (4.2)	2 (0.7)	
Hyperlipidaemia	No	55 (45.8)	178 (58.7)	0.016
	Yes	65 (54.2)	125 (41.3)	
Renal failure	No	115 (95.8)	300 (99.0)	0.031
	Yes	5 (4.2)	3 (1.0)	
Abnormal liver function	No	115 (95.8)	259 (85.5）	0.003
	Yes	5 (4.2)	44 (14.5)	
Congestive heart failure	No	88 (73.3)	235 (77.6)	0.357
	Yes	32 (26.7)	68 (22.4)	
Anaemia	No	32 (26.7)	90 (29.7)	0.534
	Yes	88 (73.3)	213 (70.3)	
Hypertension	No	32 (26.7)	141 (46.5)	*P* < 0.0001
	Yes	88 (73.3)	162 (53.5)	
History of cardiogenic shock	No	117 (97.5)	299 (98.7)	0.663
	Yes	3 (2.5)	4 (1.3)	
Myocardial infarction	No	103 (85.8)	272 (89.8)	0.25
	Yes	17 (14.2)	31 (10.2)	
Leucocytosis	No	99 (82.5)	249 (82.2)	0.938
	Yes	21 (17.5)	54 (17.8)	
Increased neutrophil numbers	No	99 (81.7)	241 (79.5)	0.489
	Yes	21 (18.3)	62 (20.5)	
Elevated uric acid	No	107 (89.2)	79 (26.1)	*P* < 0.0001
	Yes	13 (10.8)	224 (73.9)	
C-reactive protein increased >5 mg	No	82 (68.3)	113 (37.3)	*P* < 0.0001
	Yes	38 (31.7)	190 (62.7)	
Creatinine increased >200 μmol	No	113 (94.2)	281 (92.7)	0.6
	Yes	7 (5.8)	22 (7.3)	
Cardiac function grade ≥3	No	77 (64.2)	206 (68.0)	0.452
	Yes	43 (35.8)	97 (32.0)	
ASA score ≥3	No	103 (85.8)	284 (93.7)	0.009
	Yes	17 (14.2)	19 (6.3)	
Valve combined heart bypass surgery	No	112 (93.3)	292 (96.4)	0.174
	Yes	8 (6.7)	11 (3.6)	
Aortic dissection	No	114 (95.0)	287 (94.7)	1.00
	Yes	6 (5.0)	16 (5.3)	
Intraoperative blood loss >1,200ml	No	114 (95.0)	291 (96.0)	0.633
	Yes	6 (5.0)	12 (4.0)	
Aortic occlusion time >90 min	No	85 (70.8)	131 (43.2)	<0.0001
	Yes	35 (29.2)	172 (56.8)	
Blood transfusion	No	88 (73.3)	170 (56.1)	0.001
	Yes	32 (26.7)	133 (43.9)	
Operation time >5H	No	42 (35.0)	204 (67.3)	*P* < 0.0001
	Yes	78 (65.0)	99 (32.7)	
Second operation	No	116 (96.7)	300 (99.0)	*P* < 0.0001
	Yes	4 (3.3)	3 (1.0)	
Intraoperative norepinephrine administration	No	86 (71.7)	116 (38.3)	*P* < 0.0001
	Yes	34 (28.3)	187 (61.7)	
Preoperative glucocorticoid administration	No	105 (87.5)	241 (79.5)	0.56
	Yes	15 (12.5)	62 (20.5)	
Cardiopulmonary bypass time >3H	No	91 (75.8)	100 (33.0)	*P* < 0.0001
	Yes	29 (24.2)	203 (67.0)	
CPB hyperoxic state	No	62 (51.7)	207 (68.3)	0.001
	Yes	58 (48.3)	96 (31.7)	
Pulmonary disease	No	110 (91.7)	280 (92.4)	0.797
	Yes	10 (8.3)	23 (8.6%)	

AH, absence of hyperglycaemia; SIH, stress-induced hyperglycaemia.

(1) History of Cardiac Surgery: The patient had a prior history of cardiac surgery before this hospitalization. (2) The percentages primarily represent the proportion of patients included for each influencing factor related to postoperative SHG in non-diabetic patients undergoing cardiac surgery, expressed as a percentage of the total number in either the modeling or validation group. (3) All extracted influencing factors specifically refer to patients who developed SHG, indicating a potential association between these factors and the postoperative metabolic stress response in non-diabetic cardiac surgery patients. (4) Patients aged >18 years were eligible for inclusion to ensure enrollment of an adult cohort. Within this population, multivariate analysis identified age >65 years as an independent predictor of postoperative SIH in non-diabetic patients undergoing cardiac surgery.

### 3.2 Predictive nomogram for postoperative stress hyperglycemia: logistic regression vs. machine learning

**Figure 2:**
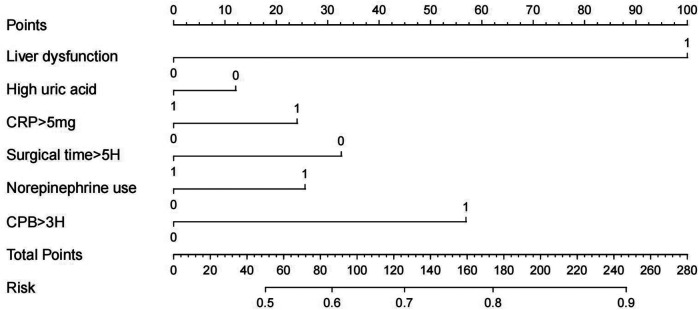
Nomogram of logistic regression model.

A training dataset comprising 423 patients was used to develop the predictive model (Figure 2). The results of the univariate logistic regression analysis are presented in Table 2. Variables that were statistically significant in the univariate analysis were included in the multivariate logistic regression model. The independent risk factors for SIH identified in this analysis included liver dysfunction, high uric acid, CRP > 5 mg, surgical time >5 h, norepinephrine use, and CPB > 3H.

**Table 2:** Multivariate analysis of non-diabetic stress hyperglycaemia after cardiac surgery.

Variable	*β*	OR	Wld*χ*^2^	95% CI	SE	*P*-value
Liver dysfunction	1.35	3.86	17.8	2.06, 7.23	0.32	<0.001
High uric acid	2.842	17.149	16.622	4.374–67.233	0.697	<0.001
CRP > 5 mg	2.016	7.508	9.936	2.144–26.300	0.64	0.002
Surgical time >5H	−1.682	0.186	7.422	0.055–0.624	0.617	<0.001
Norepinephrine use	2.486	12.008	15.344	3.462–41.648	15.344	<0.001
CPB > 3H	2.506	12.252	0.712	3.032–49.499	0.712	<0.001

CI, confidence interval; CPB, cardiopulmonary bypass; CRP, C-reactive protein; H, hours; OR, odds ratio; SE, standard error; Wld*χ*^2^, Wald chi-squared statistic.

Based on the logistic regression analysis results, a risk prediction model for the occurrence of SHG in patients without diabetes undergoing cardiac surgery was constructed by incorporating significant factors. The logistic regression equation is as follows: −4.504 + (1.35 × Liver dysfunction) + (2.842 × High uric acid) + (2.016  × CRP > 5 mg) + (−1.682 × Surgical time >5H) + (2.486 × Norepinephrine use) + (2.506 × CPB >3H). A visual nomogram was developed, with each factor assigned a specific score. The total score—the sum of all individual scores—corresponds to the probability of SHG occurrence in non-diabetic cardiac surgery patients (Figure 1).

The predictive model was evaluated using 177 patients from the validation dataset, and demonstrated strong performance, with AUC of 0.944(95% CI: 0.923–0.966), sensitivity of 0.945, and specificity of 0.943. Six key variables were identified as the most relevant for predicting SIH (Table 3).

**Table 3:** Comparison of model performance characterised by AUC, sensitivity, specificity PPV and NPV.

Model approach	AUC	Sensitivity	Specificity	PPV	NPV	F1 score
Glm	0.944 (0.923–0.966)	0.945	0.943	0.977	0.968	0.961
RF	0.877 (0.866–0.932)	0.861	0.900	0.956	0.720	0.906
GBM	0.923 (0.902–0.952)	0.934	0.716	0.892	0.811	0.912
Adaboost	0.916 (0.871–0.936)	0.881	0.850	0.936	0.739	0.908
XGB	0.904 (0.890–0.941)	0.924	0.658	0.872	0.774	0.898

AUC, area under the curve; PPV, positive predictive value; NPV, negative predictive value; RF, random forest; GBM, gradient boosting machine; Ada, adaptive boosting.

**Table 4:** Comparison of validation set characterised by AUC, sensitivity, specificity PPV, NPV and F1 score.

Model approach	AUC	Sensitivity	Specificity	PPV	NPV	F1 score
Glm	0.895 (0.848−0.942)	0.88	0.86	0.87	0.91	0.87
RF	0.780 (0.712–0.848)	0.75	0.78	0.77	0.76	0.76
GBM	0.805 (0.740–0.870)	0.78	0.79	0.79	0.81	0.78
Adaboost	0.816 (0.751–0.881)	0.80	0.81	0.80	0.83	0.80
XGB	0.795 (0.727–0.863)	0.77	0.78	0.78	0.80	0.77

### 3.3 Development of different machine learning models for SHG

**Figure 3:**
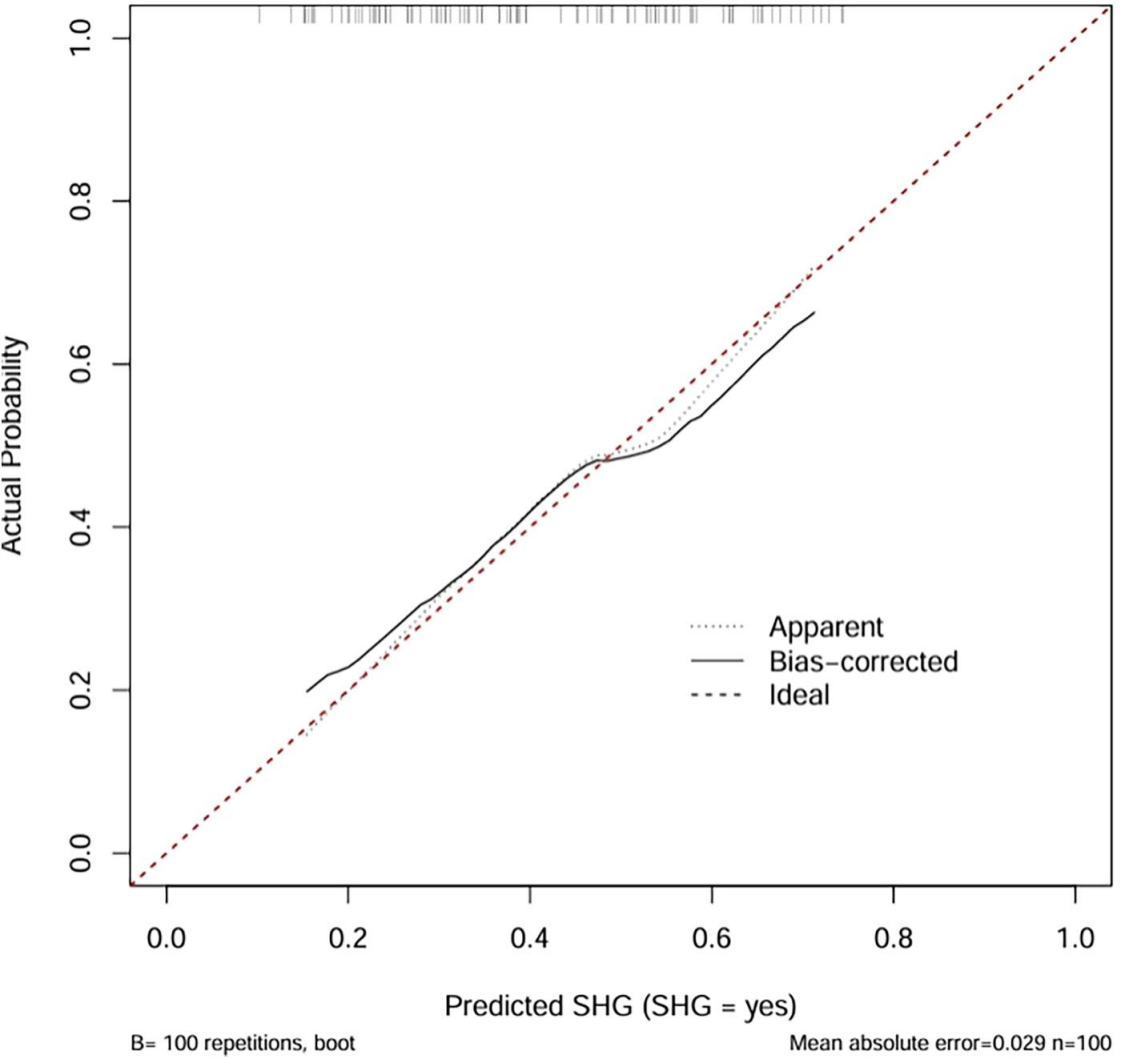
Comparison of the calibration plot for stress-induced hyperglycemia.

Five distinct machine learning algorithms were implemented to predict SHG risk in non-diabetic cardiac surgery patients: logistic regression, GBM, XGBoost, and AdaBoost (Figure 3). Model performance was rigorously evaluated through multiple metrics, including AUC-ROC, sensitivity, specificity, PPV, and NPV. Comparative analysis revealed that logistic regression achieved optimal performance, exhibiting both superior discriminative ability (highest AUC) and the most balanced sensitivity-specificity profile. Consequently, the logistic regression model was selected for external validation and subsequent development of the clinical nomogram.

### 3.4 Calibration performance of the predictive model

**Figure 4:**
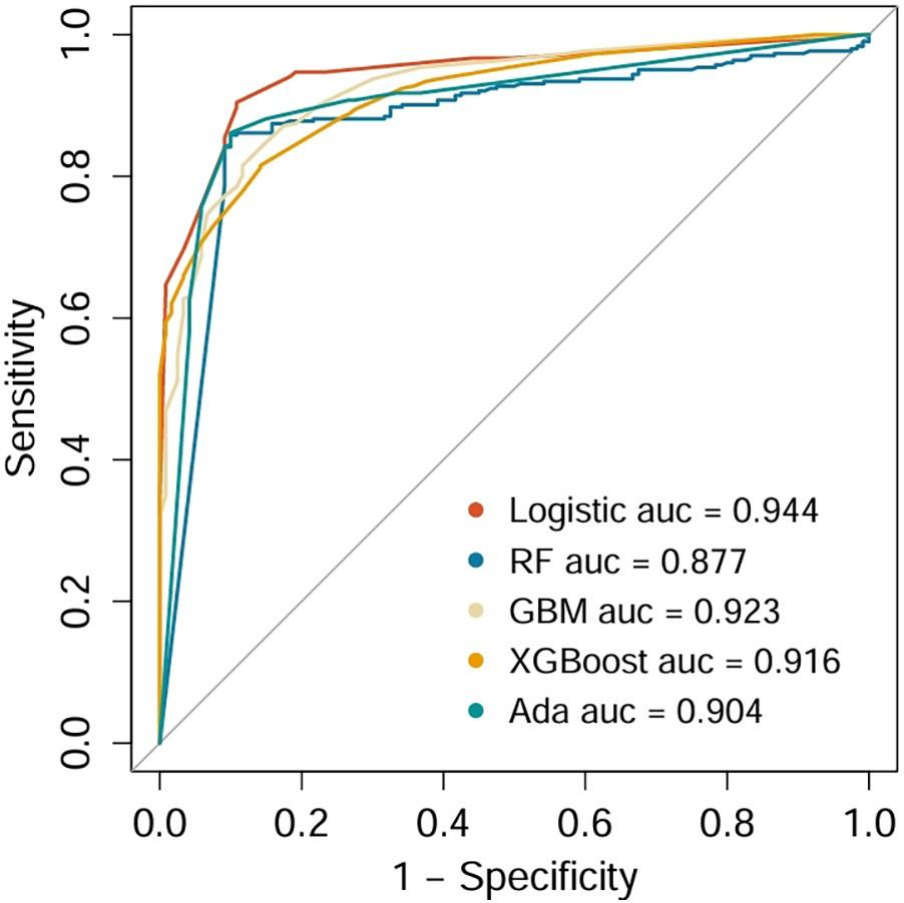
Comparison of ROCs and AUCs for prediction of SHG by the various machine learning models.

The calibration plot assessed the accuracy of predicted probabilities from the logistic regression model (Figure 4). The “Apparent” curve represents the model's performance on the training data, while the “Bias-corrected” curve, derived via bootstrap resampling (B = 100 repetitions), adjusts for overfitting. Both curves closely align with the ideal diagonal, indicating excellent agreement between predicted probabilities and observed outcomes. The minimal mean absolute error of 0.029 further confirms high calibration accuracy, suggesting that the model provides reliable probability estimates for clinical application.

The calibration curve revealed that the bias-corrected line was closely aligned with both the apparent and ideal reference lines, indicating good agreement between predicted and observed probabilities. This suggests that the model is well-calibrated and not significantly overfitted to the training data. Calibration plots are used to evaluate the accuracy of predicted probabilities in a risk prediction model. The ideal line represents perfect prediction, where the predicted risk exactly matches the observed outcome. The apparent line reflects the model's performance on the training data, while the bias-corrected line, obtained through bootstrapping, adjusts for potential overfitting and provides a more reliable estimate of the model's calibration in future samples.

### 3.5 Model performance comparison and validation in non-diabetic cardiac surgery patients

The predictive performance of five models was compared using multiple evaluation metrics, including AUC, sensitivity, specificity, PPV, and NPV. Logistic regression demonstrated the highest discriminative ability with an AUC of 0.944, with favorable sensitivity (0.945), specificity (0.943), PPV (0.977), and NPV (0.968), outperforming the more complex machine learning models. Accordingly, logistic regression was selected as the optimal model for further validation. When applied to the independent validation cohort, the model achieved an AUC of 0.895, thereby confirming its robustness, generalizability, and clinical applicability for identifying non-diabetic cardiac surgery patients at high risk for SIH.

### 3.6 Decision curve analysis of a nomogram for postoperative stress hyperglycemia

**Figure 5:**
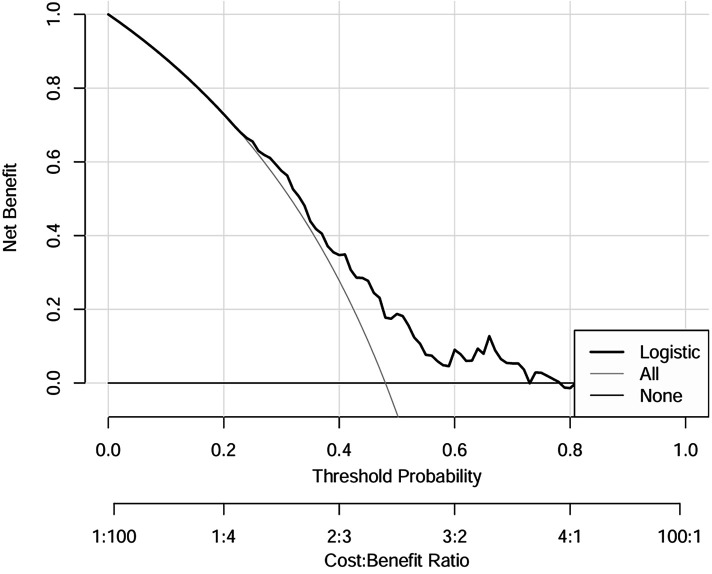
DCA of logistic regression model for the training dataset. Liver dysfunction, High uric acid, CRP > 5 mg, Surgical time >5H, Norepinephrine use, CPB > 3H. DCA, decision curve analysis.

Figure 5 presents the decision curve analysis (DCA), which evaluated the clinical utility of the nomogram for predicting SIH in non-diabetic cardiac surgery patients. The analysis framework incorporates: (1) threshold probability (*x*-axis) representing the minimum predicted risk at which clinical intervention would be considered, and (2) net benefit (*y*-axis) quantifying the trade-off between true-positive identifications and false-positive interventions across the probability spectrum. The logistic regression model demonstrated superior clinical utility, as evidenced by its consistently high net benefit across the clinically relevant threshold probability range of 20%–80%. This robust performance indicates that the model provides meaningful decision support for perioperative glycemic management in the specified risk range.

### 3.7 External validation results

In the validation cohort (Table 4), GLM model maintained the best overall performance among all tested algorithms, with an AUC of 0.895 (95% CI: 0.848–0.942), sensitivity of 0.88, specificity of 0.86, PPV of 0.87, NPV of 0.91, and F1 score of 0.87. Ensemble models, including Random Forest (AUC = 0.780), GBM (AUC = 0.805), AdaBoost (AUC = 0.816), and XGBoost (AUC = 0.795), showed moderate predictive ability. These results further confirmed the strong discriminative capacity and external generalizability of the logistic regression model, justifying its selection for the final nomogram construction.

### 3.8 Interpretable analysis of a clinical prediction model using SHAP

**Figure 6:**
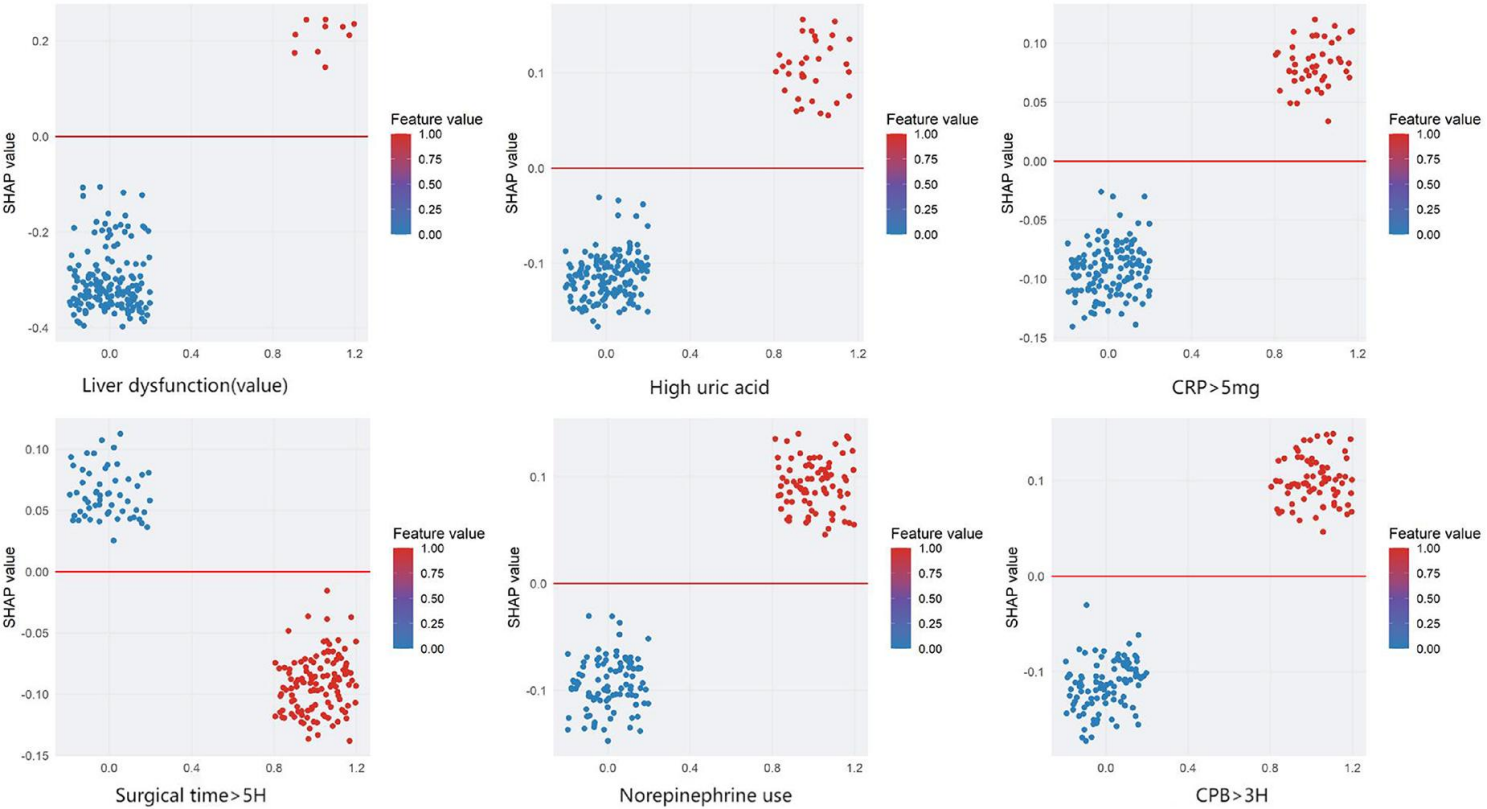
Shapley Additive exPlanations (SHAP) dependence plots for the model predicting stress hyperglycemia after cardiac surgery in non-diabetic patients. Features: Liver dysfunction, high uric acid, CRP > 5 mg, surgical time >5H, norepinephrine use, and CPB > 3H. The *x*-axis shows the SHAP value (impact on model output), and the *y*-axis lists the features. Each dot represents one patient. The color bar indicates the feature value (1 = yes, 0 = no). Positive SHAP values indicate a higher predicted risk of stress hyperglycemia, and negative values indicate a lower risk.

**Figure 7:**
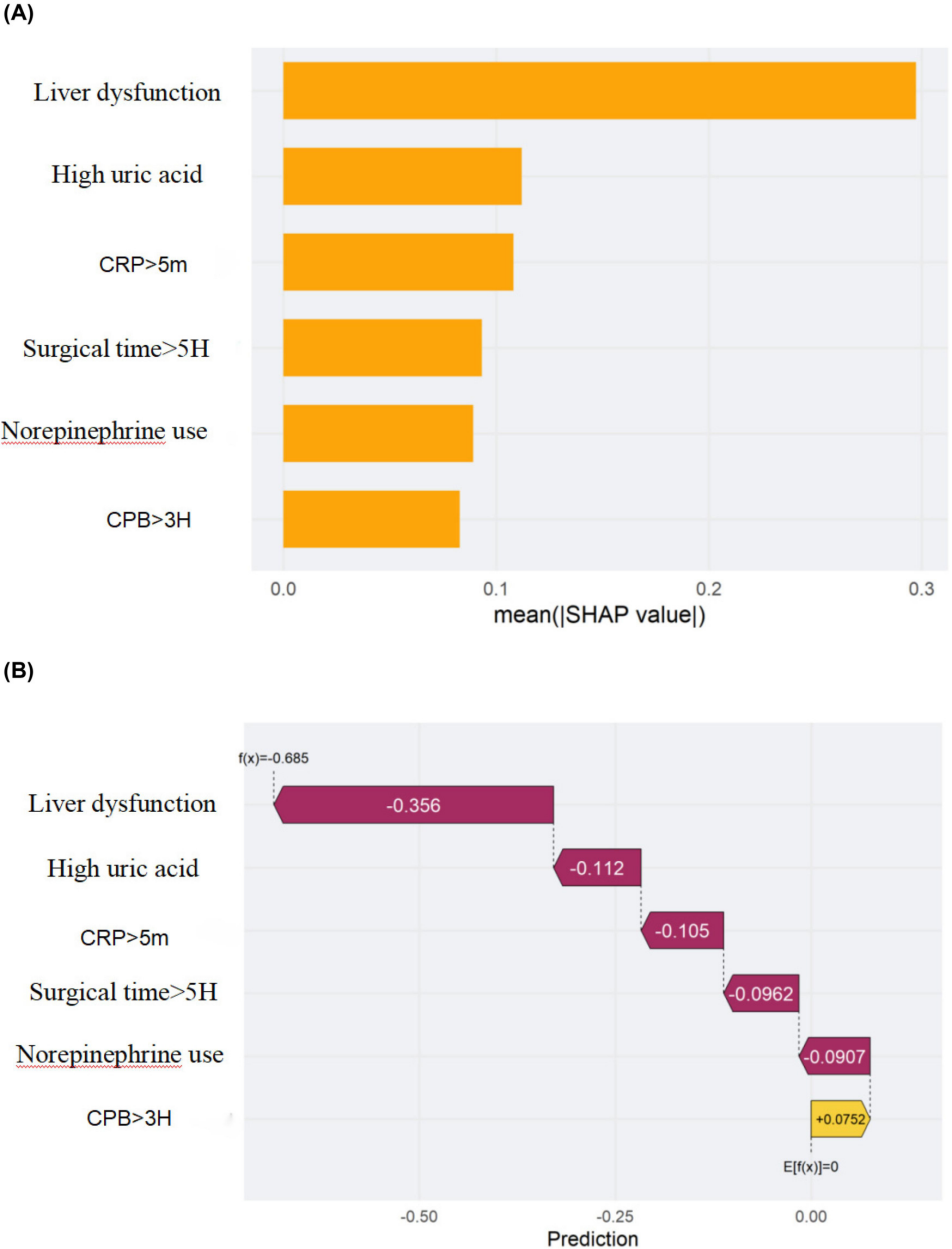
Shapley additive exPlanations (SHAP) of the machine-learning model for predicting stress hyperglycemia after cardiac surgery in non-diabetic patients. (A) Feature importance ranked by the mean absolute SHAP value across all patients. (B) SHAP decision (waterfall) plot for a representative patient showing how each feature shifts the prediction from the expected value. The *x*-axis denotes the SHAP value (impact on model output), and the *y*-axis lists the features. Each dot represents one patient in **(A)**; in **(B)** each bar is the SHAP contribution of a feature for the selected patient. Positive SHAP values indicate a higher predicted risk, whereas negative values indicate a lower risk. Feature encoding: 1 = yes, 0 = no.

We employed SHAP (SHapley Additive exPlanations) analysis to interpret the internal decision mechanisms of the prediction model. The global SHAP summary revealed that liver function indicators and hyperuricemia were the most influential features shaping model predictions. Higher liver enzyme levels were positively associated with an increased predicted risk, whereas higher, norepinephrine use,elevated C-reactive protein (CRP > 5 mg/L), and longer operative time (>5 h) showed negative SHAP contributions. Local interpretability using SHAP force plots further validated these effects by visualizing how individual feature contributions and their interactions collectively determined each patient's predicted risk. Overall, these SHAP-based findings underscore that the model's predictions are both physiologically plausible and transparent, thereby enhancing its clinical interpretability and trustworthiness (see Figures 6, 7A,B).

## 4 Discussion

In this study, we systematically developed and compared five predictive models—logistic regression, random forest, GBM, AdaBoost, and XGBoost—to identify stress-induced hyperglycemia (SIH) in 423 non-diabetic cardiac surgery patients. While previous studies have primarily focused on diabetic populations or lacked rigorous validation (26–29), research specifically addressing non-diabetic cardiac surgery patients remains limited, despite reported SIH incidence rates of 32.7%–75% in this cohort (30–32).

Notably, logistic regression outperformed more complex machine learning algorithms, achieving excellent discriminative ability (AUC = 0.944) with high sensitivity (94.5%), specificity (94.3%), positive predictive value (97.7%), and negative predictive value (96.8%). Its clinical applicability is further supported by a parsimonious model incorporating six readily obtainable perioperative variables—liver dysfunction, elevated uric acid, CRP >5 mg/L, surgical duration >5 h, norepinephrine administration, and cardiopulmonary bypass time >3 h—demonstrating robust performance in external validation (sensitivity 94.5%, specificity 93.4%). This balance of predictive accuracy and interpretability facilitates early risk stratification and individualized perioperative management, addressing a critical clinical need (33).

Several factors may explain the superior performance of logistic regression. First, the relatively small sample size may limit the ability of complex models to capture nonlinear interactions, potentially leading to overfitting. Second, the relationships between predictors and postoperative glucose appear predominantly linear, allowing logistic regression to effectively model these associations. Third, although all models except GBM were trained using default parameter settings to minimize potential optimization bias, future studies may explore whether more extensive hyperparameter tuning could reduce the performance gap between machine learning algorithms and logistic regression. Finally, logistic regression offers clear interpretability, enabling clinicians to intuitively assess each variable's contribution—a crucial advantage in clinical decision-making.

Compared with prior studies, our work provides three key advances: (1) comprehensive evaluation of five machine learning algorithms with rigorous internal and external validation; (2) identification of an interpretable model achieving optimal predictive performance; and (3) a practical, parsimonious structure facilitating early risk identification and individualized glycemic management while optimizing resource allocation.

Nevertheless, some limitations warrant consideration. The single-center, retrospective design may limit generalizability, and residual confounding cannot be excluded despite rigorous multivariate adjustment and imputation of missing data. Moreover, systematic hyperparameter tuning for complex models was not performed, which may offer further improvement in future studies.

## 5 Conclusion

In this study, we developed and validated a logistic regression model incorporating six perioperative variables—liver dysfunction, elevated uric acid, CRP >5 mg/L, surgical duration >5 h, norepinephrine administration, and cardiopulmonary bypass time >3 h—for predicting stress-induced hyperglycemia (SIH) in non-diabetic cardiac surgery patients. The model demonstrated excellent discriminative performance and consistently outperformed four advanced machine learning algorithms (random forest, GBM, XGBoost, and AdaBoost) in internal and external validation.

This work represents the first systematic comparison of multiple machine learning approaches for SIH prediction in this population, highlighting logistic regression as an optimal balance between predictive accuracy and clinical interpretability for perioperative risk stratification. Although machine learning methods are generally effective for high-dimensional datasets, inclusion of numerous predictors in a limited-sample clinical study may increase the risk of overfitting and reduce generalizability. Our variable selection process retained six clinically and statistically meaningful predictors, which captured the major risk signals of postoperative SIH while improving model robustness and interpretability.

The superior performance of logistic regression may be attributed to several factors. First, the relationships between the selected predictors and postoperative glucose levels were predominantly linear, enabling effective modeling with GLM. Second, the relatively small sample size may have limited the ability of more complex models to capture nonlinear or high-order interactions, potentially leading to overfitting. Finally, logistic regression offers clear interpretability by quantifying each variable's contribution, an important advantage in clinical contexts where transparency and actionable insights are essential. Therefore, under the specific conditions of this study, logistic regression provided a stable, reliable, and clinically applicable predictive tool for early identification of high-risk non-diabetic cardiac surgery patients.

## Data Availability

The raw data supporting the conclusions of this article will be made available by the authors, without undue reservation.
